# Synthesis and Photoluminescence Properties of LaAlO_3_: Tb^3+^ Nanophosphors Prepared by Combustion Method

**DOI:** 10.1002/bio.70579

**Published:** 2026-07-14

**Authors:** Y. Z. Halefoglu

**Affiliations:** ^1^ Department of Ceramic, Faculty of Fine Arts Cukurova University Adana Turkiye

**Keywords:** combustion method, LaAlO_3_, nanophosphor, photoluminescence, XRD

## Abstract

LaAlO_3_:Tb^3+^ nanophosphors were synthesized via a combustion method followed by calcination at 1000°C. Their structural and optical properties were characterized using XRD, FTIR, SEM, and photoluminescence analyses. XRD results confirmed the formation of a single‐phase rhombohedral perovskite structure without secondary phases, indicating successful incorporation of Tb^3+^ ions into the host lattice. FTIR analysis supported the structural stability and absence of hydroxyl‐related impurities, while SEM images revealed a porous morphology typical of combustion‐derived nanomaterials. The photoluminescence spectra exhibited characteristic Tb^3+^ emissions, with a dominant green emission at ~545 nm (^5^D_4_ → ^7^F_5_). The luminescence intensity strongly depends on Tb^3+^ concentration in the 1–7 wt% range, increasing up to 5 wt% and decreasing at higher concentrations due to concentration quenching. This behavior is attributed to nonradiative energy transfer via multipolar interactions at higher dopant levels. The enhanced luminescence is associated with improved crystallinity after high‐temperature calcination. These results demonstrate that LaAlO_3_ is a stable host for Tb^3+^ ions and highlight the importance of optimizing dopant concentration to achieve efficient green emission for potential photonic applications.

## Graphical abstract

In this study, which was conducted using the combustion method, the luminescence intensity strongly depended on Tb^3+^ concentration in the 1–7 wt% range, highlighting the critical role of activator concentration in optimizing optical performance. The strongest green emission, corresponding to the ^5^D_4_ → ^7^F_5_ transition at 545 nm, was observed at 5 wt% Tb^3+^.

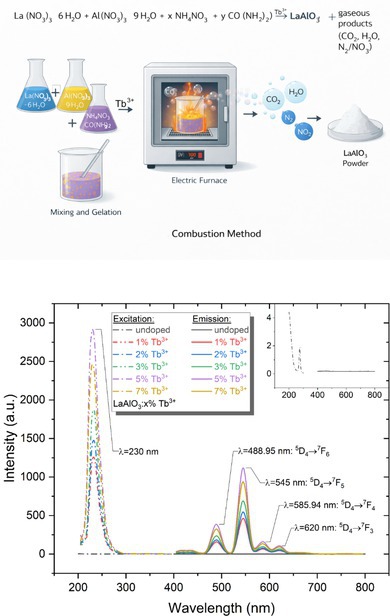

## 1 Introduction

Luminescent materials have attracted significant attention due to their broad applications in lighting, imaging, biomedical imaging, optical sensing, and solid‐state lasers. These materials absorb energy and re‐emit it as visible or near‐visible light, making them essential for advanced photonic devices [1, 2]. Luminescence involves the absorption of a higher‐energy photon followed by the emission of a lower‐energy photon after partial relaxation of the excited state. This process, known as the Stokes process, involves the dissipation of excess energy within the host matrix as lattice vibrations or heat. Although fluorescence and luminescence are often used interchangeably, they are not strictly equivalent [3].

Phosphorescence differs from fluorescence in that it involves the delayed re‐emission of absorbed photons. This delay is often attributed to electronic transitions influenced by dopant ions (e.g., transition metals) or intrinsic crystal defects such as vacancies or interstitials acting as energy traps [3].

In this context, rare earth–doped phosphors exhibit sharp emission lines, high luminescence efficiency, and excellent thermal and chemical stability. Among them, Tb^3+^‐doped LaAlO_3_ is particularly attractive due to its efficient green emission suitable for optoelectronic applications. Rare earth–activated perovskites such as LaAlO_3_:Tb^3+^ are particularly attractive due to their sharp 4f–4f transitions and structural stability, enabling better understanding of dopant–host interactions. Extensive research has led to significant progress in recent years. LaAlO_3_:Tb^3+^ nanophosphors offer strong application potential due to their low‐cost raw materials and chemical stability [2, 4].

The trivalent terbium ion is widely used as an activator in green nanophosphors. Understanding the optical properties of Tb^3+^ is crucial for its technological applications [5, 6]. Compared with other perovskite hosts such as YAlO_3_ [7], the spectroscopic properties of LaAlO₃ remain underexplored and not yet fully characterized. Although several studies have reported the structural and luminescent properties of Tb^3+^‐doped LaAlO₃ [5, 8, 9], most focus on bulk ceramics or materials prepared by conventional solid‐state methods. A systematic investigation of combustion‐derived LaAlO₃:Tb^3+^ nanophosphors remains limited, particularly in understanding how synthesis and annealing conditions influence crystallinity, defect concentration, and luminescence. Previous studies have also explored only narrow dopant ranges without comprehensive correlation analysis. This work aims to address these gaps by synthesizing LaAlO₃:Tb^3+^ nanophosphors via a rapid combustion method and systematically correlating their structural, morphological, and optical properties. In contrast to previous studies that primarily focused on emission characteristics, this study explores the relationship between microstructure and luminescence behavior.

In contrast, this study systematically investigates combustion‐derived LaAlO₃:Tb^3+^ nanophosphors over a wide dopant concentration range (1–7 wt%), enabling a systematic evaluation of concentration‐dependent luminescence. Williamson–Hall (W–H) analysis is employed to quantify crystallite size and lattice strain, and these parameters are correlated with photoluminescence behavior. In addition, structural, morphological, and spectroscopic analyses are integrated to clarify the role of the microstructure in emission properties. The concentration quenching mechanism is further interpreted using critical distance (*R*_c_) analysis, providing insight into the dominant energy transfer processes. LaAlO₃ crystallizes in a rhombohedral structure (space group R3c) at room temperature (300 K). When heated above 530°C, it transforms into a cubic structure (space group Pm3m). Within the lattice, La^3+^ ions occupy sites with D₃ symmetry, and rare earth ions substitute for these positions [10, 11].

Luminescence in Tb^3+^‐doped LaAlO₃ is strongly influenced by crystallographic structure, oxygen stoichiometry, and annealing conditions. Defect‐related emission near 730 nm decreases as Tb^3+^ ions occupy vacancy sites. Increasing annealing temperature reduces defects, improves crystallinity, and shifts emission from violet (^5^D₃) to dominant green (^5^D₄ → ^7^F₅), with maximum intensity at 900°C–1000°C. However, the mechanisms governing the interplay between defect‐related and activator‐centered emission remain unclear. This study focuses on the optical response of samples annealed at 1000°C [5, 8, 9].

In this study, LaAlO₃ nanophosphors doped with 1–7 wt% Tb^3+^ were synthesized via a solution combustion method, and their morphological and spectroscopic properties were analyzed. NH₄NO₃ and urea were used as oxidizer and fuel, respectively. Key synthesis parameters, including ignition temperature and fuel‐to‐oxidizer (F/O) ratio, were optimized to obtain phase‐pure, homogeneous, and porous materials [5, 8, 12].

To address these limitations, this study aims to (i) synthesize LaAlO₃:Tb^3+^ nanophosphors via a combustion method, (ii) investigate the effect of Tb^3+^ concentration (1–7 wt%) on structural and microstructural properties, and (iii) correlate these parameters with photoluminescence behavior to identify the optimal activator concentration.

## 2 Materials and Methods

### 2.1 Synthesis and Doping of LaAlO₃ Nanocrystals

LaAlO₃:Tb^3+^ nanophosphors doped with 1–7 wt% Tb^3+^ were synthesized using a combustion method. X‐ray diffraction (XRD), Fourier transform infrared (FTIR), and scanning electron microscopy (SEM) analyses were conducted to confirm the formation of the perovskite structure. All reactions were conducted using high‐purity reagents (Sigma‐Aldrich): La (NO₃)₃·6H_2_O (≥99.9%), Al (NO₃)₃·9H_2_O (≥98%), Tb (NO₃)₃·5H_2_O (99.9%), and ammonium nitrate (NH₄NO₃; ≥99.9%). Urea served as the fuel (reducing agent), while NH₄NO₃ and metal nitrates acted as oxidizers within the redox combustion system. The target oxide material was obtained via a controlled solution combustion process, in which metal nitrates served as oxidizers in a stoichiometrically balanced fuel–oxidizer system [13]. Stoichiometric amounts of La (NO₃)₃·6H_2_O and Al (NO₃)₃·9H_2_O were weighed based on the calculated redox balance between fuel and oxidizer components. Urea and ammonium nitrate were then added in a 1:1 ratio and the mixture was further ground to ensure homogeneity. The combustion reaction was completed within 2–3 min at 550°C, producing a porous foam‐like precursor, which was subsequently recalcined at 1000°C for 2 h [14].

Considering the oxidizing valence of La (NO₃)₃·6H_2_O, Al (NO₃)₃·9H_2_O, Tb (NO₃)₃·5H_2_O, and NH₄NO₃, together with the reducing contributions of urea, the stoichiometric fuel‐to‐oxidizer (F/O) ratio was calculated to be 1.0 for all compositions. The fuel‐to‐oxidizer ratio was determined using the propellant chemistry approach, where the total oxidizing valence of nitrate groups [from La (NO₃)₃, Al (NO₃)₃, Tb (NO₃)₃, and NH₄NO₃] and the reducing valence of urea [CO (NH_2_)_2_] were calculated. In this method, carbon and hydrogen act as reducing elements, while oxygen supplied by nitrate groups contributes to the oxidizing valence. For a stoichiometric combustion reaction, the total oxidizing valence must be equal to the total reducing valence. Accordingly, the F/O ratio was adjusted to unity (F/O = 1), ensuring complete combustion and maximum heat release with any residual reactants. This approach is widely used in solution combustion synthesis to achieve thermodynamic balance and homogeneous product formation. Due to the multicomponent nature of the combustion system and the evolution of multiple gaseous products, writing a fully balanced reaction equation in a simple closed form is not straightforward. Therefore, the reaction is expressed in a generalized form, while stoichiometric balance is ensured through the fuel‐to‐oxidizer ratio calculation described above. During combustion, the primary reaction product was phase‐pure LaAlO₃, while CO_2_, H_2_O, and NO_*x*_ were released as gaseous by‐products:

$$\begin{array}{c} \mathrm{La}\;\left(\mathrm{NO}₃\right)₃\cdot {\text{6H}}_{2}O+\mathrm{Al}\;\left(\mathrm{NO}₃\right)₃\cdot {\text{9H}}_{2}O+x\;\mathrm{NH}₄\mathrm{NO}₃\\ +y\;\mathrm{CO}\;{\left({\mathrm{NH}}_{2}\right)}_{2}⟶\text{LaAlO}₃+\text{gaseous products}\;\left({\mathrm{CO}}_{2},H_{2}O,N_{2}/{\mathrm{NO}}_{x}\right) \end{array}$$

Due to the complex redox nature of the combustion process and the evolution of multiple gaseous species, the reaction is expressed in a generalized form. The stoichiometry of the system was controlled through fuel‐to‐oxidizer ratio calculations based on total oxidizing and reducing valences.

XRD analysis was first performed to verify the formation of LaAlO₃. After confirming a single‐phase perovskite structure, Tb^3+^ doping was introduced during heating under stirring using the same synthesis parameters. To evaluate the effect of dopant concentration, samples with Tb^3+^ contents ranging from 1 to 7 wt% were prepared. The doped LaAlO₃ nanocrystals exhibited strong green emission, with maximum luminescence efficiency at 1000°C [5, 13]. Annealing temperature significantly influenced both morphology and structural order.

### 2.2 Characterization of the Nanophosphor Materials

In this study, the synthesized nanophosphor material was characterized using various analytical techniques. The crystalline structure and phase composition were analyzed by XRD using a Philips PW‐1710/00 diffractometer with CuKα radiation (*λ* = 1.5418 Å) at room temperature. XRD patterns were collected over a 2*θ* range of 10°–90° with a step size of 0.5 s and a scan rate of 7°/min, under operating conditions of 40 kV and 40 mA. Data analysis was performed using Xpowder software to obtain detailed crystallographic information.

FTIR spectra were recorded using a Jasco FT/IR‐6700 spectrometer equipped with a Michelson interferometer (28°) and corner‐cube mirrors. The movable mirror was supported by a frictionless mechanical bearing and driven by an electromagnetic coil. Mirror positioning was monitored via a He–Ne laser with AccuTrac digital signal processing (DSP) to ensure high accuracy. The spectrometer used a KRS‐5 window with a Ge/KBr beam splitter and a DLATGS detector, covering the spectral range of 7800–350 cm^−1^. This setup enabled a precise identification of vibrational modes and functional groups in the samples.

Photoluminescence measurements were performed using a Photon Technology International (PTI) QuantaMaster 30 spectrophotometer. The system includes pulsed and continuous xenon lamps, Czerny–Turner monochromators, and sensitive detectors, enabling both steady‐state and time‐resolved measurements. Excitation and emission spectra were recorded at room temperature under ambient conditions. The monochromator slit widths were set to 5 nm for both excitation and emission to optimize spectral resolution while maintaining signal intensity.

The surface morphology and microstructure were examined using field‐emission SEM. The microscope operates at accelerating voltages ranging from 100 V to 30 kV, with a probe current of 100 nA and magnification from 6× to 1,000,000×. In high‐vacuum mode, the resolution reaches 1.2 nm at 30 kV and 2.3 nm at 1 kV, while in low‐vacuum mode, it is 2.0 nm at 30 kV. The system includes multiple detectors (InColumn SE, BSE, 4‐segment BSE, STEM, GSED, and low‐vacuum Balong with an energy‐dispersive X‐ray spectrometer (EDXS)) for elemental analysis and a navigation camera for precise sample positioning.

## 3 Results and Discussion

### 3.1 XRD Analysis

**FIGURE 1:**
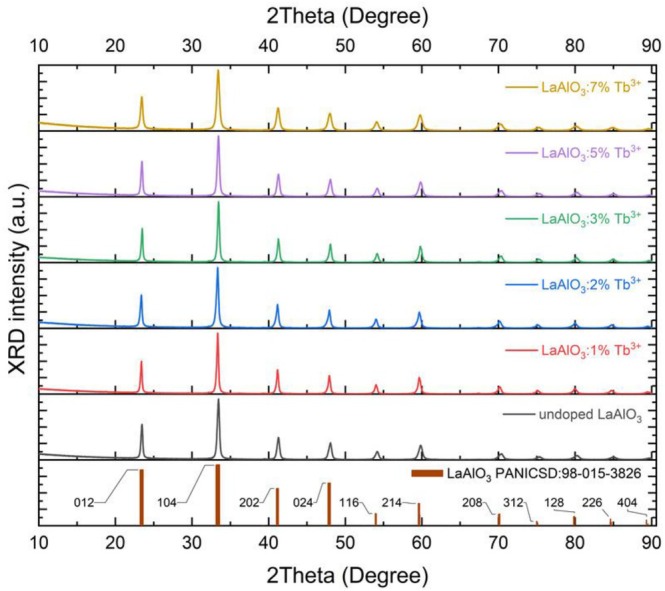
XRD patterns of undoped and Tb^3+^‐doped LaAlO₃ nanophosphors (1–7 wt% Tb^3+^) synthesized by the combustion method and calcined at 1000°C. All diffraction peaks are indexed to the rhombohedral perovskite phase (space group R–3c), confirming phase purity and successful incorporation of Tb^3+^ ions without the formation of secondary phases.

XRD analysis was performed to identify crystalline phases, confirm the incorporation of Tb^3+^ ions into the LaAlO₃ lattice, and evaluate structural changes induced by doping and annealing (Figure 1). This method also allows the identification of optimal synthesis conditions. Tb^3+^ doping levels between 1 and 7 wt% were systematically studied. The results show that Tb^3+^ incorporation does not produce secondary phases or structural transitions within the detection limits of the instrument [15, 16].

All diffraction peaks of undoped and Tb^3+^‐doped LaAlO₃ samples (1–7 wt%) were indexed to the rhombohedral perovskite phase (space group R–3c), consistent with the standard reference pattern (PDF No. 98‐015‐3826). The peak positions and relative intensities agree with previous reports [5, 8, 13], confirming the formation of phase‐pure rhombohedral LaAlO₃. The main diffraction peaks appear at 22.9°, 32.4°, 39.7°, 46.8°, and 52.8°, corresponding to the (012), (104), (202), (024), and (116) planes, respectively. Additional reflections at 57.3°, 67.1°, 76.4°, and 82.8° further support the rhombohedral phase. The refined lattice parameters (*a* ≈ 5.363 Å, *c* ≈ 13.104 Å) agree with reported values for bulk LaAlO₃, indicating that Tb^3+^ ions are incorporated at La^3+^ sites without significant lattice distortion. No significant peak broadening or splitting is observed, supporting the formation of a homogeneous solid solution. This structural integrity is essential for achieving a well‐defined local environment for efficient radiative transitions.

**FIGURE 2:**
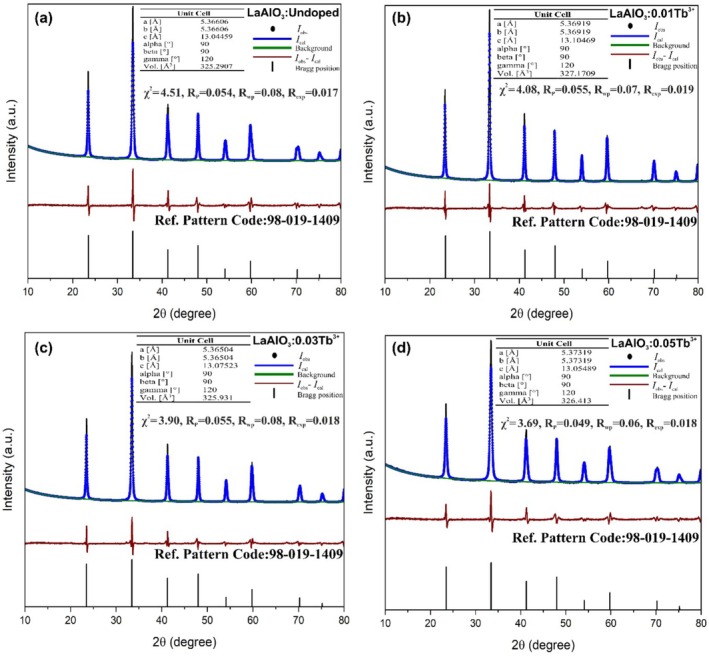
Rietveld‐refined XRD patterns of LaAlO₃:Tb^3+^ nanophosphors with varying Tb^3+^ concentrations: (a) undoped, (b) 0.01, (c) 0.03, and (d) 0.05. The observed and calculated patterns are shown along with the difference profiles (*I*_obs_ − *I*_cal_), and the vertical tick marks indicate the Bragg reflection positions.

In order to provide a more quantitative structural analysis, profile fitting and lattice parameter refinement were performed for all compositions. The refined XRD patterns (Figure 2) show good agreement between the observed and calculated profiles, as indicated by the low reliability factors (*R*_p_, *R*_wp_, and *χ* ^2^). The refined unit cell parameters are summarized in Table 1.

**TABLE 1:** Refined structural parameters of LaAlO₃:Tb^3+^ nanophosphors obtained from Rietveld refinement of XRD patterns.

LaAlO_3_				
Unit cell	Undoped	0.01Tb^3+^	0.03Tb^3+^	0.05Tb^3+^
*a* (Å)	5.36606	5.36919	5.36504	5.37319
*b* (Å)	5.36606	5.36919	5.36504	5.37319
*c* (Å)	13.04459	13.10469	13.07523	13.05489
alpha, beta, gamma (°)	90, 90, 120	90, 90, 120	90, 90, 120	90, 90, 120
Vol. (Å^3^)	325.2907	327.1709	325.931	326.413
*χ* ^2^	4.5127	4.0833	3.9004	3.6996
*R*_p_	0.0548	0.0553	0.0523	0.0498
*R*_wp_	0.0801	0.0799	0.0735	0.0680
*R*_exp_	0.0177	0.0195	0.0188	0.0183

*Note:* The lattice constants (*a*, *b*, *c*), unit cell volume, and refinement reliability factors (*χ* ^2^, *R*_p_, *R*_wp_, and *R*_exp_) are listed for different Tb^3+^ concentrations.

The results reveal that all samples maintain the rhombohedral perovskite structure (space group R–3c) without forming secondary phases. A systematic variation in lattice parameters was observed with increasing Tb^3+^ concentration, confirming the successful incorporation of Tb^3+^ ions into the LaAlO₃ lattice. This analysis goes beyond qualitative phase identification and provides quantitative insight into the structural evolution as a function of dopant concentration.

A slight but systematic shift of diffraction peaks toward higher 2*θ* values is observed with increasing Tb^3+^ concentration. This shift is attributed to the substitution of La^3+^ ions (ionic radius: 1.36 Å, CN = 12) by smaller Tb^3+^ ions (1.27 Å, CN = 12), resulting in a slight unit cell contraction. This behavior is consistent with lanthanide contraction and confirms that Tb^3+^ ions occupy La^3+^ lattice sites rather than forming separate Tb‐containing phases. The peak narrowing in samples annealed at higher temperatures is attributed to grain growth and improved crystallinity, in agreement with Scherrer's equation. For samples annealed above 900°C, the crystallite size increases significantly, correlating well with the enhanced luminescence properties discussed later.

**FIGURE 3:**
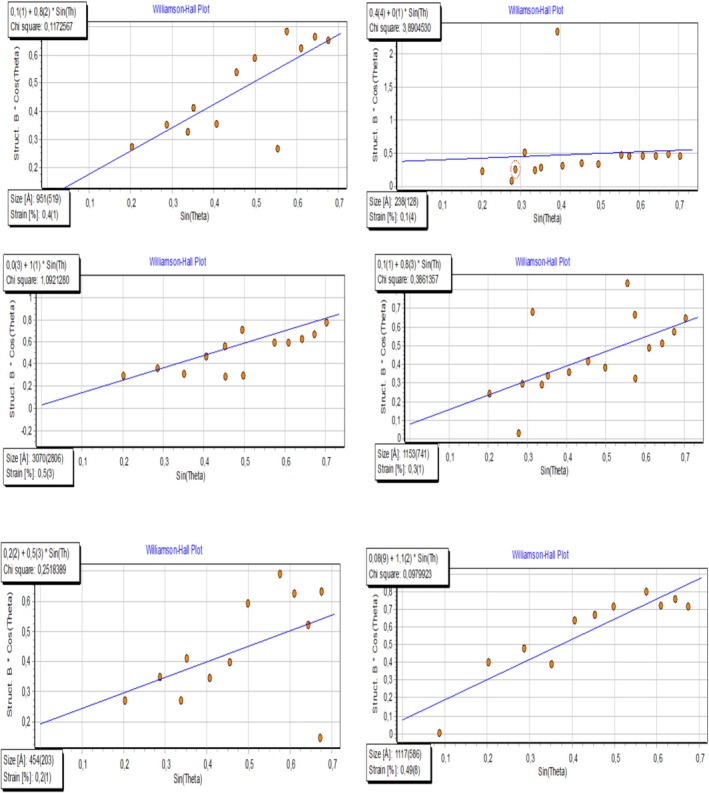
Williamson–Hall (W–H) plots of undoped and Tb^3+^‐doped LaAlO₃ nanophosphors. The linear fitting of βcos*θ* versus 4sin*θ* allows separation of crystallite size and lattice strain contributions, revealing the influence of Tb^3+^ doping on microstructural evolution.

Figure 3 shows the Williamson–Hall (W–H) plots for undoped and Tb^3+^‐doped LaAlO₃ nanophosphors (1–7 wt%). The W–H analysis reveals a nonmonotonic variation in crystallite size and lattice strain with Tb^3+^ concentration. The undoped sample exhibits a crystallite size of ~95 nm and a relatively high microstrain (~0.4%). At 1 wt% Tb^3+^, the crystallite size decreases to ~23 nm, accompanied by a reduction in strain (~0.1%), indicating suppression of grain growth and partial strain relaxation. At higher dopant levels (e.g., 2 wt%), the crystallite size increases (~307 nm) along with strain (~0.5%), reflecting competing effects of lattice distortion and grain growth. Overall, these results demonstrate that Tb^3+^ incorporation modifies the balance between crystallite growth and lattice strain in LaAlO₃.

To validate the W–H results, crystallite sizes were also estimated using the Scherrer equation for the (104) reflection at 2*θ* ≈ 33°, and the results are summarized in Table 2. The discrepancy between the W–H and Scherrer values arises from their different assumptions: the Scherrer method considers only size‐induced broadening, whereas the W–H approach accounts for both crystallite size and lattice strain. Therefore, in the presence of microstrain, the W–H method provides a more comprehensive description of peak broadening.

**TABLE 2:** Crystallite size (*D*) and microstrain (*δ*) values of LaAlO₃ and Tb^3+^‐doped LaAlO₃ nanophosphors (0–5 wt%) calculated using the Debye–Scherrer method based on the (104) diffraction peak at 2*θ* ≈ 33°.

Concentration	LaAlO_3_	Undoped	0.01Tb^3+^	0.03Tb^3+^	0.05Tb^3+^
Debye–Scherer	*D* (nm)	51.238	65.679	59.181	41.116
	δ × 10^−3^ (nm^−2^)	0.3809	0.2318	0.2855	0.5915

The observed nonmonotonic trend suggests that microstructural evolution is governed by competing mechanisms. At low Tb^3+^ concentrations, lattice distortion may hinder crystallite growth, whereas at higher concentrations, defect accumulation and thermally activated processes promote grain growth. Consequently, peak broadening is attributed to the combined effects of finite crystallite size and lattice microstrain.

**FIGURE 4:**
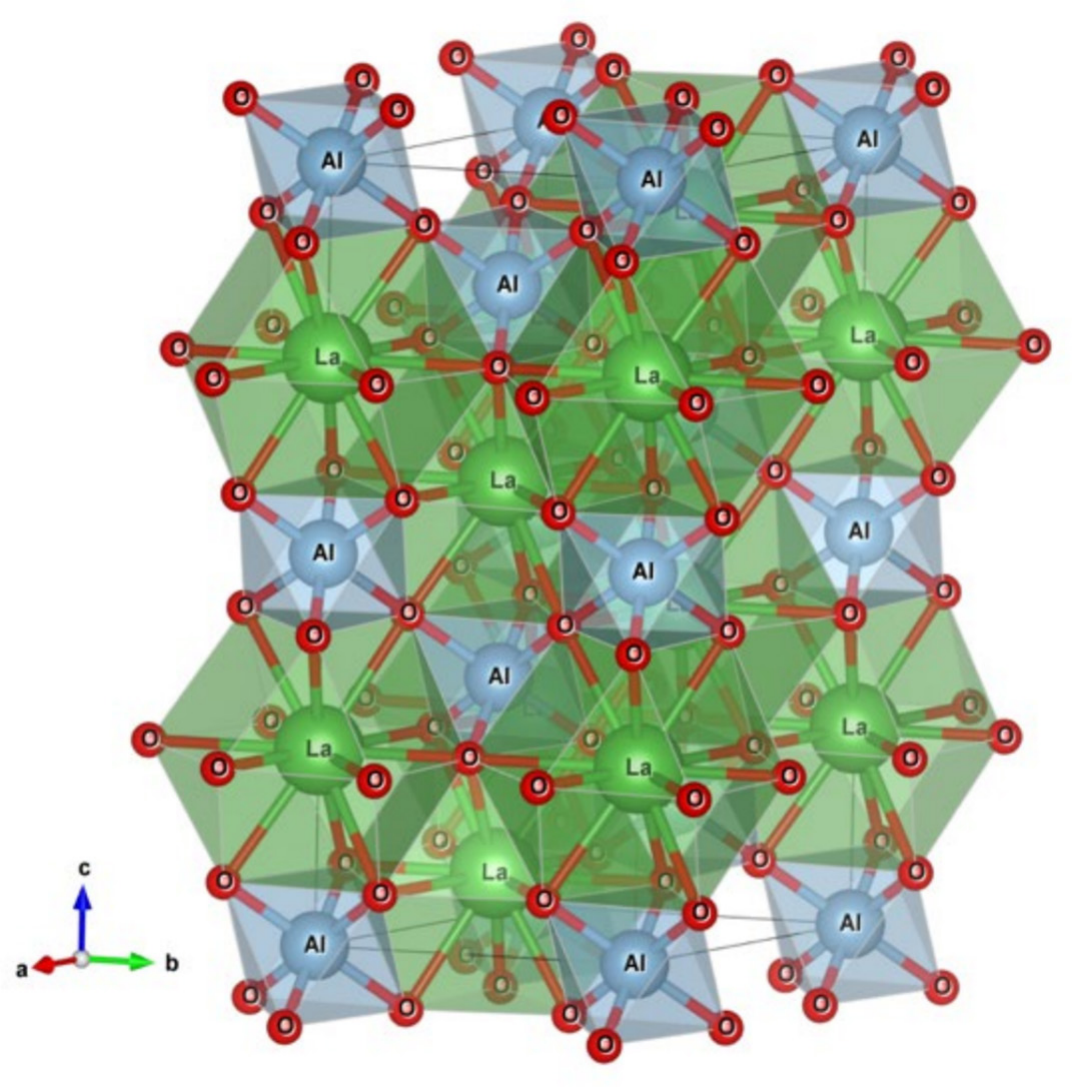
Schematic representation of the rhombohedral perovskite structure of LaAlO₃, illustrating the A‐site (La^3+^/Tb^3+^) and B‐site (Al^3+^) cation positions and the corner‐sharing AlO₆ octahedral framework.

In the LaAlO₃ perovskite structure (Figure 4), La^3+^ ions occupy the A‐sites, while Al^3+^ ions are located at the centers of AlO₆ octahedra (B‐sites), forming a three‐dimensional framework. Oxygen ions are shared at the corners of these octahedra, forming a three‐dimensional perovskite framework. Tb^3+^ ions occupy La^3+^ A‐sites due to their similar ionic radius, preserving the AlO₆ octahedral framework. As a result, the rhombohedral symmetry is maintained, and no secondary phases are observed in the XRD patterns even at higher Tb^3+^ concentrations. Only minor peak shifts are detected, confirming the stable dopant incorporation.

In the rhombohedral R–3c structure, AlO₆ octahedra form a corner‐sharing network, with symmetry arising from cooperative octahedral tilting. La^3+^ ions at the 12‐coordinated A‐sites are replaced by Tb^3+^ ions, which are comparable in size to La^3+^ (1.36 Å). This substitution does not significantly alter octahedral tilting or overall symmetry, preserving the rhombohedral structure. The small ionic radius mismatch minimizes lattice strain, consistent with the absence of peak splitting and only slight peak broadening. A‐site substitution modifies the local crystal field, influencing 4f–4f electronic transitions and photoluminescence behavior. The absence of significant distortion or oxygen nonstoichiometry ensures a structurally stable host lattice and a uniform environment for efficient radiative processes.

According to the Hume–Rothery rules, the large ionic radius difference between Tb^3+^ and Al^3+^ favors substitution at the A‐site rather than the B‐site. This preserves the AlO₆ framework and rhombohedral symmetry, while modifying the local crystal field and thus the 4f–4f transitions responsible for photoluminescence.

### 3.2 FTIR Analysis

**FIGURE 5:**
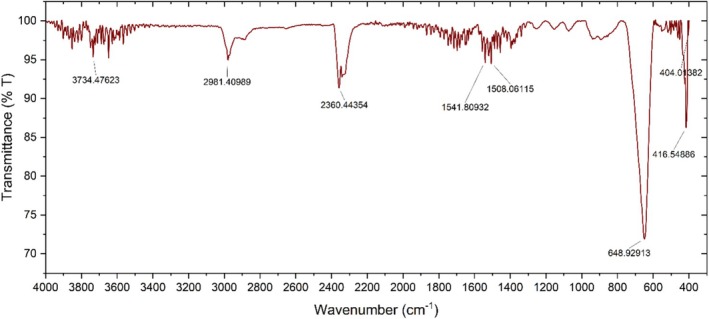
FTIR spectra of LaAlO₃:5wt%Tb^3+^ nanophosphors (4000–400 cm^−1^), showing characteristic vibrational modes associated with Al–O stretching and La–O–Al bending, confirming the formation of the perovskite structure and the absence of significant impurity phases.

Figure 5 shows the FTIR spectrum of LaAlO₃:5wt%Tb^3+^ nanophosphors in the 4000–400 cm^−1^ range. The band at ~648 cm^−1^ corresponds to Al–O stretching vibrations of the AlO₆ octahedra, while the band near 416 cm^−1^ is attributed to La–O–Al bending modes characteristic of the rhombohedral LaAlO₃. These assignments agree with the previous reports for LaAlO₃ structures [8].

The absence of the broad O–H stretching band (3400–3430 cm^−1^) indicates minimal hydroxyl content, confirming the effective removal of moisture during calcination [8, 14, 17, 18, 19].

The weak band near 2360 cm^−1^ is attributed to atmospheric CO_2_ and is not related to the sample composition. A weak band in the 1540–1500 cm^−1^ region may arise from residual nitrate or carbonate species. The absence of additional impurity bands further supports the formation of phase‐pure LaAlO₃.

No significant peak shifts or new bands are observed upon Tb^3+^ doping, indicating that the host lattice is preserved. These results confirm the structural compatibility of Tb^3+^ within the LaAlO₃ lattice and are consistent with the XRD analysis [20].

### 3.3 SEM Analysis

**FIGURE 6:**
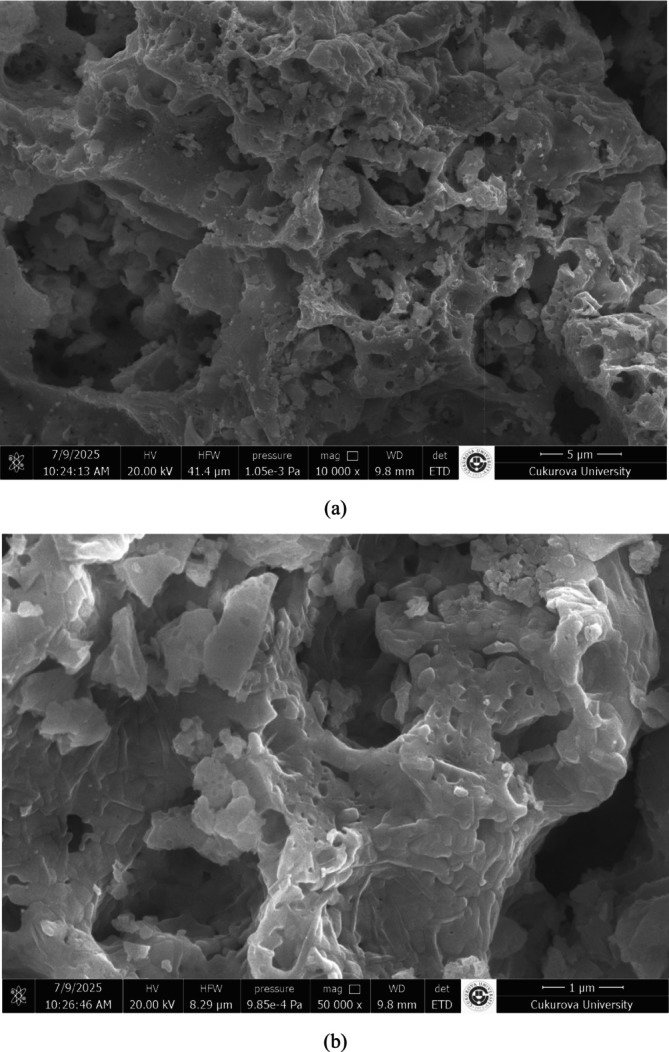
SEM micrographs of LaAlO₃:Tb^3+^ nanophosphors synthesized by the combustion method and calcined at 1000°C: (a) low‐magnification image showing the highly porous, foam‐like morphology generated by rapid gas evolution during combustion; (b) high‐magnification image revealing loosely connected primary nanoparticles embedded within the porous framework.

Figure 6a,b show SEM micrographs of LaAlO₃:Tb^3+^ nanophosphors at different magnifications. The particles exhibit a porous, foam‐like morphology composed of loosely connected primary grains. This structure is characteristic of combustion synthesis and results from rapid gas evolution during precursor decomposition, which limits particle coalescence and grain growth. As a result, nanoscale particles are distributed within a porous framework, enhancing surface area and light scattering, which are beneficial for luminescence performance. The relatively uniform grain size distribution suggests a stable combustion process with a well‐balanced fuel‐to‐oxidizer ratio. These observations are consistent with previous reports indicating that combustion conditions influence microstructure and optical properties [13, 16].

A precise particle size distribution analysis is challenging due to the highly porous and interconnected morphology of the combustion‐derived powders, in which clear particle boundaries are difficult to distinguish. However, qualitative evaluation of the SEM micrographs indicates that the material consists of nanoscale primary particles that form larger agglomerates.

The observed morphology suggests that the primary particle size is in the nanometer range, which is consistent with the crystallite size estimated from XRD analysis using the Scherrer method (Table 2). These primary particles are strongly agglomerated, forming a three‐dimensional porous network. The agglomeration behavior can be attributed to the high surface energy of nanosized particles and the rapid gas evolution during the combustion process.

This type of agglomeration can be classified as *soft agglomeration*, referring to loosely bound particle clusters formed without significant necking or sintering between adjacent particles. Such an open and porous morphology may be beneficial for phosphor applications, as it can enhance light scattering and increase the effective interaction volume, potentially improving luminescence performance. However, excessive agglomeration may also introduce nonradiative pathways depending on defect density and particle connectivity.

### 3.4 Energy‐Dispersive X‐Ray Analysis

**FIGURE 7:**
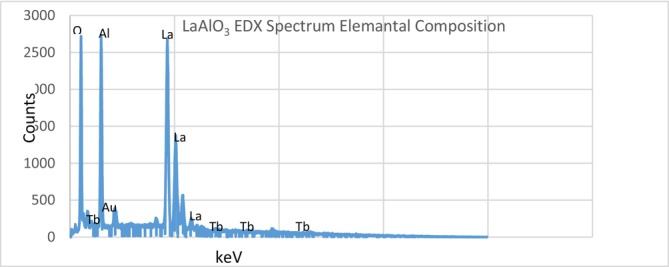
EDX spectrum of the LaAlO₃:5wt%Tb^3+^ nanophosphor showing the elemental composition of the sample. Characteristic peaks of La, Al, O, and Tb confirm the successful incorporation of Tb^3+^ into the LaAlO₃ host lattice, while the Au signal originates from the sputter coating applied prior to SEM‐EDX analysis.

The elemental composition of the LaAlO₃:5 wt% Tb^3+^ nanophosphor was analyzed using EDX spectroscopy, and the results are presented in Table 3 together with the spectrum shown in Figure 7. The spectrum clearly confirms the presence of La, Al, O, and Tb elements, verifying that all expected constituents are present in the synthesized material. The semiquantitative EDX analysis indicates that the elemental composition is approximately La (61.83 wt%), Tb (3.25 wt%), Al (12.56 wt%), and O (22.35 wt%). The detection of Tb, despite its relatively low concentration and weak peak intensity, provides direct evidence of successful dopant incorporation into the LaAlO₃ lattice. The discrepancy between the nominal Tb content (5 wt%) and the measured value (~3.25 wt%) arises from the normalization of elemental composition over the entire LaAlO₃ matrix, as well as the intrinsic limitations of EDX analysis. In particular, EDX is less sensitive to light elements such as oxygen and may exhibit reduced accuracy for low‐concentration dopants due to interaction volume effects and surface sensitivity. Therefore, the obtained values should be considered as semiquantitative rather than absolute. The Au peak observed in the spectrum originates from the gold sputter coating applied prior to SEM‐EDX analysis and is not related to the sample composition. Although elemental mapping was not performed, the absence of secondary phases in the XRD patterns and the uniform morphology observed in SEM images suggest a homogeneous distribution of Tb^3+^ ions within the LaAlO₃ host lattice. The consistency between the EDX results and the XRD analysis further confirms that Tb^3+^ ions are successfully incorporated into the LaAlO₃ lattice without forming secondary phases.

**TABLE 3:** Quantitative elemental composition of LaAlO₃:5 wt%Tb^3+^ nanophosphors obtained from the EDX spectrum.

Element	Weight %	Atomic %	Net Int.
O K	19.66	53.77	1211.38
Al K	16.49	26.75	1391.66
La L	55.74	17.56	2062.88
Tb L	2.22	0.61	57.38
Au L	5.89	1.31	53.64

### 3.5 Photoluminescence Analysis

**FIGURE 8:**
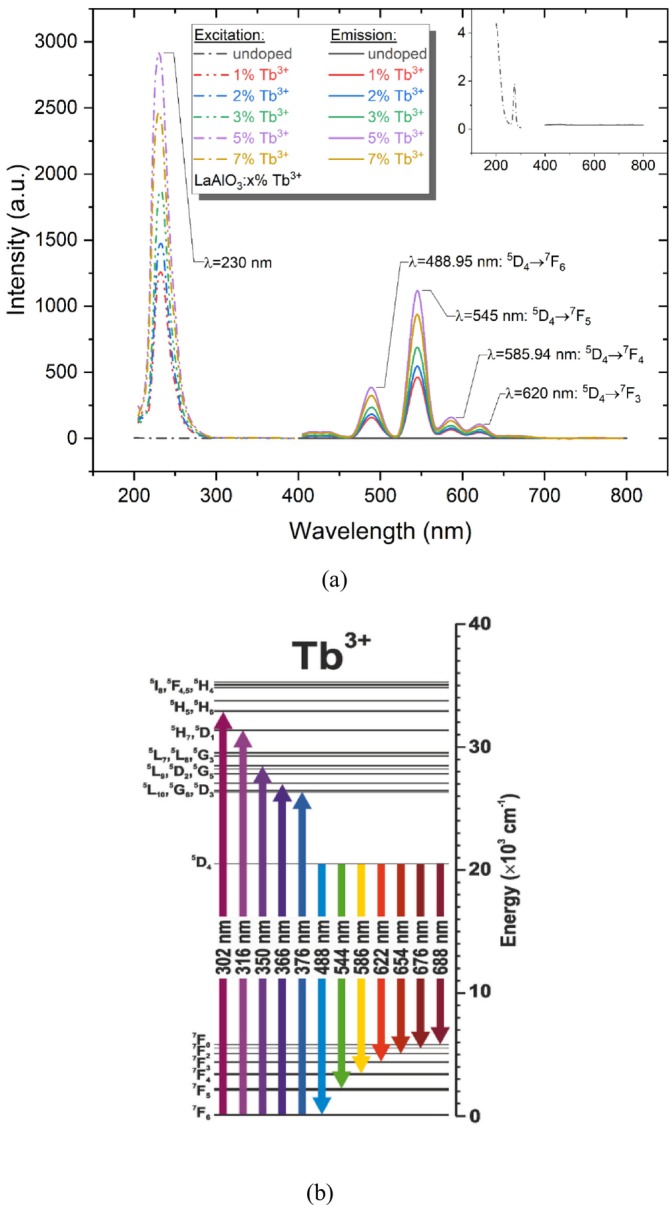
(a) Photoluminescence emission spectra of LaAlO₃:Tb^3+^ nanophosphors (1–7 wt% Tb^3+^) calcined at 1000°C, showing the characteristic green emission (^5^D₄ → ^7^F₅) with maximum intensity at optimal doping concentration. (b) Energy level diagram of Tb^3+^ ions illustrating excitation, relaxation, and energy transfer processes responsible for luminescence and concentration quenching.

Figure 8a shows the photoluminescence spectra of LaAlO₃:Tb^3+^ nanophosphors calcined at 1000°C. All samples exhibit characteristic Tb^3+^ emission lines arising from the ^5^D₄ → ^7^F_J transitions, with the strongest emission at ~545 nm (^5^D₄ → ^7^F₅), responsible for green luminescence. The excitation spectrum (monitored at 545 nm) shows a broad host absorption band below 300 nm and sharp 4f–4f transitions of Tb^3+^ ions, indicating efficient energy transfer to the ^5^D₄ level of Tb^3+^ ions. Additional emission peaks at 488 nm (^5^D₄ → ^7^F₆), 585 nm (^5^D₄ → ^7^F₄), and 620 nm (^5^D₄ → ^7^F₃) are also observed, consistent with previous reports [5, 8, 9, 21].

The energy‐level diagram (Figure 8b) illustrates the luminescence mechanism. Upon excitation, electrons populate the ^5^D₃ and ^5^D₄ levels, followed by nonradiative relaxation from ^5^D₃ to ^5^D₄, making ^5^D₄ the dominant emissive level. At low to moderate Tb^3+^ concentrations (1–5 wt%), the increasing number of optically active centers enhances emission intensity. At higher concentrations (>5 wt%), nonradiative energy transfer between neighboring Tb^3+^ ions becomes significant, leading to concentration quenching. As a result, the emission intensity peaks at 5 wt% and decreases at higher dopant levels.

The enhanced luminescence at 1000°C is attributed to improved crystallinity, which reduces lattice defects and suppresses nonradiative recombination, as confirmed by XRD analysis. The efficient population of the ^5^D₄ metastable level is attributed to the favorable crystal‐field environment around Tb^3+^ ions, leading to strong ^5^D₄ → ^7^F_J radiative emission. A decrease in luminescence at 7 wt% Tb^3+^ is attributed to concentration quenching [22].

To further clarify the concentration‐quenching behavior, the critical distance between neighboring Tb^3+^ ions (*R*_c_) was calculated using Blasse's expression:

$$R_{c}=2{\left(\frac{3V}{4\pi \mathrm{xZ}}\right)}^{1/3}$$

where *Z* is the number of activator sites per unit cell, *V* is the unit‐cell volume, and *x* is the optimal Tb^3+^ concentration. For LaAlO₃:Tb^3+^ nanophosphors, *Z* = 4, *V* = 54.39 Å^3^ (from XRD refinement), and *x* = 0.02, yielding an *R*_c_ of ≈ 8 Å. Because this value is much larger than the 4–5 Å threshold, the quenching mechanism cannot originate from exchange interactions. Therefore, the decrease in emission intensity above 5 wt% Tb^3+^ is attributed to long‐range multipolar interactions, most plausibly dipole–dipole coupling, which is the dominant mechanism in rare earth–doped perovskites. A log–log intensity‐concentration analysis, typically used to identify the interaction mechanisms, was not performed due to the limited number of dopant concentrations; however, the *R*_c_ value supports a multipolar quenching mechanism.

In order to further elucidate the energy transfer mechanism responsible for concentration quenching, it is important to consider the interaction between neighboring Tb^3+^ ions. In rare earth–doped systems, energy transfer between activator ions can occur via exchange interactions or multipolar interactions, depending on the critical distance (*R*_c_). Since the calculated *R*_c_ value (~8 Å) is significantly larger than 4 Å, exchange interaction can be excluded, and the dominant mechanism is attributed to multipolar interactions.

Multipolar interactions, including dipole–dipole, dipole–quadrupole, and quadrupole–quadrupole mechanisms, are generally considered in rare earth–doped systems. In the present case, dipole–dipole interaction is the most probable mechanism due to the nature of 4f–4f transitions and the relatively large interionic distance. At higher Tb^3+^ concentrations, the reduced distance between neighboring ions enhances nonradiative energy transfer, where excitation energy migrates between Tb^3+^ ions and is eventually dissipated at quenching centers, leading to decreased emission intensity. Based on the estimated critical distance (*R*_c_ ≈ 8 Å), which exceeds the threshold for exchange interactions (~4 Å), the concentration quenching is attributed to long‐range multipolar interactions, predominantly dipole–dipole coupling.

Due to the limited dopant range, a detailed log–log multipolarity analysis was not performed; however, the *R*_c_ value provides sufficient evidence for the proposed mechanism.

At higher dopant concentrations, nonradiative energy transfer processes become dominant, promoting energy migration to defects or impurity sites and reducing emission intensity. This behavior is consistent with the observed luminescence trend, with maximum emission at ~5 wt% Tb^3+^. Overall, the luminescence behavior is governed by the balance between radiative and nonradiative processes as a function of dopant concentration.

## 4 Conclusion

LaAlO₃:Tb^3+^ nanophosphors were successfully synthesized via a combustion method followed by calcination at 1000°C. The structural analysis confirmed a single‐phase rhombohedral perovskite with Tb^3+^ incorporation at the A‐sites. Photoluminescence results showed strong green emission (~545 nm, ^5^D₄ → ^7^F₅) with a clear dependence on Tb^3+^ concentration. Emission intensity increased up to 5 wt% Tb^3+^ and decreased at higher concentrations due to multipolar quenching. These results highlight the critical roles of dopant concentration and calcination temperature in tuning luminescent properties. Improved crystallinity correlates with enhanced emission intensity, emphasizing the importance of thermal treatment. LaAlO₃ is a stable and efficient host for Tb^3+^ activators. Future work should focus on co‐doping, defect engineering, and integration into LED prototypes for practical applications.

## Author Contributions

**Y. Z. Halefoglu:** conceptualization, investigation, funding acquisition, writing – original draft, writing – review and editing, visualization, validation, methodology, software, formal analysis, project administration, resources, supervision, data curation.

## Funding

The author has nothing to report.

## Conflicts of Interest

The author declares no conflicts of interest.
